# Covariation of Branch Lengths in Phylogenies of Functionally Related Genes

**DOI:** 10.1371/journal.pone.0008487

**Published:** 2009-12-29

**Authors:** Wai Lok Sibon Li, Allen G. Rodrigo

**Affiliations:** 1 Bioinformatics Institute, University of Auckland, Auckland, New Zealand; 2 Department of Computer Science, University of Auckland, Auckland, New Zealand; 3 School of Biological Sciences, University of Auckland, Auckland, New Zealand; University of California San Diego, United States of America

## Abstract

Recent studies have shown evidence for the coevolution of functionally-related genes. This coevolution is a result of constraints to maintain functional relationships between interacting proteins. The studies have focused on the correlation in gene tree branch lengths of proteins that are directly interacting with each other. We here hypothesize that the correlation in branch lengths is not limited only to proteins that directly interact, but also to proteins that operate within the same pathway. Using generalized linear models as a basis of identifying correlation, we attempted to predict the gene ontology (GO) terms of a gene based on its gene tree branch lengths. We applied our method to a dataset consisting of proteins from ten prokaryotic species. We found that the degree of accuracy to which we could predict the function of the proteins from their gene tree varied substantially with different GO terms. In particular, our model could accurately predict genes involved in translation and certain ribosomal activities with the area of the receiver-operator curve of up to 92%. Further analysis showed that the similarity between the trees of genes labeled with similar GO terms was not limited to genes that physically interacted, but also extended to genes functioning within the same pathway. We discuss the relevance of our findings as it relates to the use of phylogenetic methods in comparative genomics.

## Introduction

Estimating lineage-specific substitution rates and divergence dates has become an increasingly important aspect of the reconstruction of evolutionary history [1]–[4]. Differences in substitution rates from lineage to lineage have been attributed to variation in neutral rates of substitution, population size, generation times, and selective forces. These together are responsible for the non-ultrametric distances on a tree [5], [6] and gives rise to lineage-specific variation in molecular evolutionary rates.

More recently there has been focus on the possibility of lineage-gene-specific differences in substitution rate [7], [8]. The number of substitutions acquired by a protein-coding gene may increase during periods of rapid adaptive change or decrease because of strong structural or functional constraints on the coded protein. The molecular evidence for such specific selection-mediated substitutions has been the subject of much research since the pioneering paper of Messier and Stewart [9], [10]–[14]. These selection-mediated substitutions are by definition non-neutral and therefore would not be expected to be consistent across genes or across lineages.

The proteins that genes encode do not function individually but rather within entire pathways, though this is usually ignored in models of genic evolution [15]. In fact, it is reasonable to suggest that natural selection acts on a group of genes that collectively perform a biological function. Under the presence of selection, both functional and structural constraints will be expected to cause the divergence rates of functionally-related genes to covary.

Physically interacting genes are known to co-evolve, in the sense that there are correlated rates of substitution between genes of interacting proteins [16]–[20]. The way proteins function as physical structures can constrain the mutations that are allowed to persist. This is particularly evident in protein domains involved in direct physical interactions with other proteins, where protein interaction may fail if mutations that change the protein structure occur at the site of interaction. Correlated substitutions that occur within a species lineage can result in similarities in substitution rates across species. In addition to this, different lineages undergo different extents of selection pressure for any given biological function. Due to this effect of coevolution, the selection pressures applied to a function are reflected on many or all the genes involved in that function. These two effects in combination have been shown to cause the coevolution of genes [21], [22].

Accordingly, there is resemblance in branch lengths in the gene trees of interacting protein coding genes [23]. Pazos and Valencia [24] were the first to use this observed pattern of coevolution across species to predict the interaction between genes. In their study, they were able to predict pairwise interaction of gene products with 79% accuracy in the dataset used [25]. Other approaches to predicting gene interactions using coevolution have also been devised that utilize methods similar to Pazos and Valencia [21], [26]–[32].

We argue here that coevolution and similarities in substitution rates across species are not limited purely to interacting gene pairs. Our hypothesis differs from that of Fryxell's [23] in that we suggest a more general evolutionary relationship: coevolution occurs not only specifically amongst genes that interact with each other but also amongst genes that are known to be involved in the same biological function. Coevolution is partially driven by similarity in selective pressures acting on functionally related genes [33]. Also, as all genes that interact ultimately form a network in metabolic pathways, it is expected that some “contagious” correlation will extend to functionally related genes. Our argument is supported by recent studies, which show that there is correlation in patterns of evolution amongst genes involved in related biological processes [21], [33]–[39]. In particular, recent studies by Juan et al. [21] have found patterns of coevolution across genes from the interactomes of the NADH-quinone oxidoreductase complex and the flagellar assembly machinery, though the study did not explicitly state whether or not direct physical interactions occurred between these genes.

Though our hypothesis is supported by literature in theory and results, it has been found that genes operating within the same pathway can vary in selective pressures. A study by Rausher et al. [40] and its follow up study by Lu et al. [41] have demonstrated that differing selection pressures occur between upstream and downstream genes of the anthocyanin pathway in the *Ipomoea* genus. Hence it should be noted that correlation in evolutionary rates does not necessarily occur amongst genes in all pathways.

The aim of our study was to find how the correlation in branch length varies across the different biological functions. This matter is particularly important for phylogenetic inference and studies of comparative genomics. In particular, we aimed to determine whether the similarities in gene tree branch lengths that are seen in genes that have physically interacting gene products also exist between genes that are functionally related. As a comparison to Rausher et al.'s results, we attempt to determine whether the mode of selection is common within the different pathways in our set of species. In our study, we found that there is a correlation in branches lengths of genes trees from functionally related genes that do not necessarily have physical interactions. Results show that the degree of correlation varies greatly across different biological functions. We also discuss the findings of our study towards gene choice when computing species divergences.

## Materials and Methods

The aim of our study is to predict the relationship between genes that are functionally related. We hypothesize that correlation between genes can be used to infer the function when the function of some genes in a correlated set is known. The species phylogeny is used here as a basis to detect changes in substitution rate across lineages.

### Visualizing Substitution Patterns amongst Genes and Lineages As a Matrix

First we consider a new scheme of visualizing variation in substitution rates amongst genes and lineages which uses a matrix of gene tree branch lengths. Consider a collection of orthologous genes from a set of species. If the true species topology is known and assumed to be the same for all genes, all the gene trees can be built with the topology constrained. This results in a set of genes trees with the same branches but optimized to have gene-specific branch lengths. We can consider a matrix, *B*, of dimensions *M*×N, where *M* is the number of genes, and N is the number of branches on the tree, N (N is equal to 2*n*-3 in an unrooted tree, where *n* is the number of taxa). Each entry *B_ij_* of the matrix represents the length of branch *j* in gene tree *i*. It should be noted that the order of branches and genes in the matrix is arbitrary, but constant across all genes.

### Matrix Transformation

The first step of our analysis procedure is to transform the branch lengths to allow for our models to take into account global species-specific effects (e.g. the faster rate of evolution on the lineages of mice and rats compared to larger longer-lived mammals such as humans). We introduce a procedure to transform within the matrix notation. The procedures described here are analogous to standard procedures used in data transformation in microarray analysis [42].

In this procedure, all zero branch lengths are replaced with the minimum non-zero value in the matrix. In the analysis of our dataset, the lower bound of zero was never reached. All values of the matrix were then log transformed. The empirical distribution of branch lengths across all genes for a particular branch tends to be significantly skewed. An example of this is shown in Figure S1, where this distribution can be seen clearly. Matrix entries are therefore log transformed to obtain values that are less skewed.

### Generalized Linear Models

We use Generalized Linear Models (GLM) as a method to predict the function of a gene by its evolution pattern. A GLM is a least squares regression method that uses a link function to model the relationship between sets of independent random variables and the response variable. Binary functions can be modeled by comparing the value predicted by the GLM to cut-offs which determine whether or not the observation is predicted to be involved in the process. A range of cut-off values can be iterated through to control for different false positive and false negative error rates.

In our case, the independent random variables are from rows of the matrix *B'*, where each variable corresponds to the normalized length of a branch for a given gene tree. The response variable was a binary variable representing whether the gene was involved in a particular biological function. Specifically, we are testing whether each gene is involved in the respective function. By using individual binary GLMs to model each biological function, each gene can be classified as being involved in multiple functions. *Probit* was used as the link function.

An advantage of using GLMs as our method of identifying correlation is that the method automatically takes into account variation within the same variable. Thus, the method will take into account any variation within a given branch across all the genes, such as effects from the natural species distances.

### Dataset Compilation

We take our dataset from that used in Pazos et al. [25] which consists of amino-acid alignments of *Escherichia coli* genes against orthologs in other prokaryotic species. Pazos et al. obtained these alignments by BLASTing [43] of the *E. coli* protein sequences against the genomes of other prokaryotic species. Pazos et al. included in the dataset the top hits that have an E-value above a chosen cut-off point.

As the number of species included increases, the number of genes that are homologous between all the species decreases. We wanted to choose a set of species that not only allowed for a reasonable number of branches in the gene trees, but also had a sufficient number of orthologous genes. We chose our species set by finding the ten species that were most frequently present in Pazos et al.'s dataset and took the gene alignments that contained all ten species (*Bacillus subtilis*, *Mesorhizobium loti*, *Caulobacter crescentus*, *Escherichia coli*, *Salmonella typhi*, *Salmonella typhimurium*, *Yersinia pestis*, *Pasteurella multocida*, *Vibrio cholerae* and *Pseudomonas aeruginosa*).

### Recovering Species and Gene Tree Topologies

As the dataset consists of prokaryotes, gene tree topologies can differ from the species topology as a result of horizontal gene transfers (HGT). To filter out genes where the gene relationship may not reflect the underlying species relationships, MCMC analyses were performed using MrBayes [44]. For each of the genes, we computed two runs, each with one cold chain and three heated chains, under a mixed amino-acid model with four gamma (*γ*) rate categories and allowing invariable sites (*i*). Prior distributions of tree branch lengths and the gamma shape parameter were set to exponential distributions with *λ* = 10 and the starting tree was set to random. The chains were run for 1100000 steps and sampled every 200 steps, with the first 500 trees discarded.

The posterior distributions were taken and used to determine the correct relationships amongst the species. Probabilities of each tree topology from the 95% credible set of trees was taken for each gene. The probabilities of each topology for each gene were multiplied to get the joint posterior probability of each topology over all genes, assuming independence of genes. The tree with the highest joint posterior probability was chosen as the best estimate of phylogeny. The procedure here is justified by the fact that if the tree priors for each gene are assumed to be equal, and the genes are unlinked, then this calculation is monotonic with the joint posterior probability, as follows. The posterior probability of a given tree, τ, over all genes, *D_i_*, is:
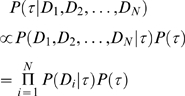
(1)


If the posterior probabilities are obtained separately for each gene then:
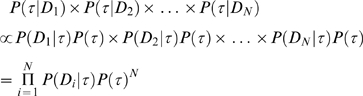
(2)


As can be seen, Eqn (2) is monotically (but non-linearly) proportional to Eqn (1).

When a particular topology is not found in a gene, a minimum probability is assigned, equivalent to one divided by the number of samples taken in the MCMC analysis. According to this criterion, the most probable tree topology yielded a log probability of −2289.62. In contrast, the second most probable tree had a log probability of −2814.34. The most probable species topology found from our MCMC analysis concurs with the one used in Pazos et al.'s study, which is derived from neighbor-joining trees of distances in the 16S rRNA gene.

As the issue of HGT needed to be addressed, any genes that had significant uncertainty as to whether they had the species topology were filtered from the dataset. Genes were excluded if the MrBayes analysis did not contain the species topology we found to be the most probable within the 95% credible set of trees. As a result, 222 genes out of 471 were excluded from the dataset.

### Dataset Annotation

Gene Ontology (GO) [45] annotations on biological processes and molecular functions that the *E. coli* genes are involved in were obtained from the UniProt [46] and iProClass [47] databases. iProClass contains functional annotations that were electronically determined. These annotations are determined by high sequence similarity to genes of known function in other species. These annotations were used to increase the amount of annotation for our gene set, as there are insufficient annotations in *E. coli* that have been experimentally identified. Genes containing no GO annotation for known process or function were removed from our dataset. All GO terms used took into account exact synonyms for the same term. The resulting dataset contained alignments of 219 homologous genes from the 10 prokaryotic species.

For every possible combination of GO biological process and molecular function, we found the number of genes that were involved in both GO terms. We use pairings of GO process and function here as a representation of distinct biological functions. Our justification for this is that using only one of biological process or molecular function will group together genes that are not necessarily functionally related. Each gene was labeled with the process-function pairs that it is involved in. This information is later used in training and benchmarking GLMs of each function. We filtered out process-function pairs that had less than 7 genes involved because training models with a low number of positive cases can lead to biased and badly fit models [48]. An assumption made here is that the biological function of each gene is identical across the species in the alignment.

### Algorithm Implementation

Our program was written in Java 1.5 and utilizes some of the functions and classes from the Phylogenetic Analysis Library (PAL) package version 1.5 [49].

### Phylogenetic Analysis

Each of the gene trees were constructed by maximum likelihood with PHYML 3.0 [50]. Gene tree topologies were constrained to the species topology that we found previously. A Dayhoff + γ + i model with 8 relative substitution rate categories was used [51] Equilibrium amino-acid frequencies, proportion of invariable sites and distribution shape were estimated from sequence data of each gene.

## Results

A leave-one-out test was used to benchmark the accuracy of the GLMs. We constructed GLMs, each time training the models with all but one of the genes. The trained models were applied to get a numerical prediction of the excluded gene. This was repeated with each of the genes in the dataset. The predictions from the GLMs were converted to estimates of whether the gene is involved in a process for a range of cut-off values. This was carried out for each of the process-function pairs to obtain the overall prediction accuracy of each term pair. As measures of accuracy, false positive error rates, true positive error rates and the Receiver Operator Characteristics (ROC) area under the curve were calculated with the ROCR package in R [52].

**Figure 1:**
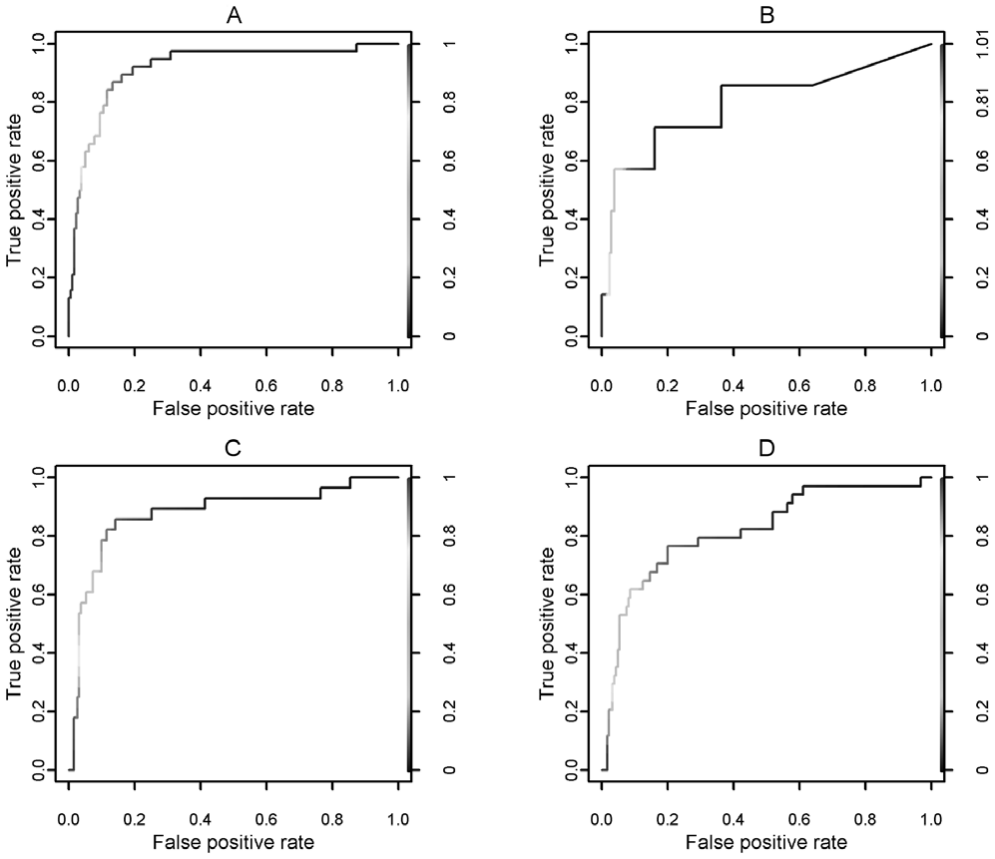
Plots of true positive rate against false positive rate for a few example GO process-function pairs. The predictions from the GLMs of each function were estimated using different values of the cut-off point (shown by the colored scale on the right), and error rates calculated from these predictions. (a)–(d) shows the accuracy of four related ribosomal functions within the GO process of “translation”. The four GO functions are “structural constituent of ribosome”, “tRNA binding”, “rRNA binding” and “RNA binding”, respectively.

Table 1 shows a list of process-function pairs used and the accuracy of the models as assessed by ROC area. For values of ROC area, an area of 0.5 indicates that the classifier performs randomly. In contrast, an area of 1.0 would be achieved by a perfect classifier. It can be seen from Table 1 that the ROC areas for classification appears to vary greatly across the terms. There appears to be good correlation in genes that are identified as being involved in both the GO process of “translation” and GO function of “structural constituent of ribosome”, with a ROC area of 0.92 when trying to predict the function of these genes. From Figure 1a, it can be seen that the false positive rate of predicting gene involvement in this particular function was in general very low across.

**Table 1:** Prediction accuracy of the GLMs for the leave-one out tests, measured by the ROC area under the curve.

GO biological process(es)	GO molecular function(s)	Number of genes	ROC area	Adjusted *p*-value
translation	structural constituent of ribosome	38	0.92	0.00
translation	rRNA binding	28	0.88	0.00
translation	RNA binding	34	0.82	0.00
translation	tRNA binding	7	0.80	0.01
translation	protein binding	22	0.69	0.03
translation	aminoacyl-tRNA ligase activity; ATP binding; ligase activity	12	0.71	0.05
regulation of transcription, DNA-dependent	protein binding	8	0.70	0.10
transport	protein binding	7	0.69	0.10
protein folding	protein binding	7	0.66	0.17
DNA replication	protein binding	8	0.67	0.19
tRNA aminoacylation for protein translation	aminoacyl-tRNA ligase activity; ATP binding; ligase activity; nucleotide binding	7	0.62	0.23
DNA repair	hydrolase activity	8	0.61	0.23
translation	nucleotide binding	15	0.59	0.23
response to DNA damage stimulus	hydrolase activity	7	0.55	0.37
transport	ATP binding	7	0.54	0.41
DNA replication	DNA binding	7	0.52	0.41
SOS response	DNA binding	7	0.49	0.52
metabolic process	transferase activity	10	0.48	0.58
regulation of transcription, DNA-dependent	RNA binding	7	0.43	0.67
metabolic process	protein binding	7	0.39	0.74
metabolic process	catalytic activity	13	0.42	0.74
DNA repair; response to DNA damage stimulus	DNA binding	11	0.41	0.74
cell cycle; cell division	nucleotide binding	8	0.38	0.74
DNA repair; response to DNA damage stimulus	ATP binding; nucleotide binding	7	0.33	0.80
transcription	DNA binding	7	0.29	0.86
transcription	protein binding	8	0.25	0.91

Different GO process terms and function terms often shared the exact same set of genes. For example the functions of “aminoacyl-tRNA ligase activity”, “ATP binding” and “ligase activity” within the “translation” process have the same genes involved in them. These are grouped as a single category in the table.

The accurate prediction also extends to genes that are identified as being in other ribosomal related functions within “translation”, with ROC areas of 0.80, 0.88 and 0.82 in “tRNA binding”, “rRNA binding” and “RNA binding” (“RNA binding” is a generalization of both types of RNA), respectively (Figure 1b–d). Upon closer inspection, these four “translation” related RNA functions contain genes that overlap, such that the genes involved in one of the functions were often involved in some of the others. This ROC area indicates low correlation between the trees of genes annotated as being involved in this process. In contrast, for a majority of the process-function pairs, correlation in gene tree branch lengths was not seen between genes identified as having the same GO terms, with the GLMs performing approximately at random.

Randomization tests were carried out to determine whether the high correlations in our processes-function pairs are statistically significant. For each pair, a null distribution of 1000 sample replicates was constructed. Each replicate was generated by randomly selecting genes in the dataset to be involved in a null biological function. The number of genes selected to be involved in the null function in each replicate is equivalent to the number of genes involved in the process-function term. A leave-one-out test was carried out on each of the replicates and the ROC area calculated. From these randomizations the *p*-values of obtaining the actual ROC areas for each GO term combination were calculated. *p*-values were adjusted with false discovery rate correction [53] to correct for multiple comparisons (shown in Table 1). It can be seen that the correlation observed in the ribosomal functions of translation was highly significant to a 5% error rate. This indicates that the high ROC areas produced by these gene grouping were unlikely caused by sampling effects. Apart from the translation related functions, there were no other functions that were significant.

As a control, we tested whether the accuracy of the prediction was directly correlated to the number of genes that were used to train the models. It is a known issue in statistics that under-trained models with too few cases of each class produce biased and inaccurate predictions. We computed a linear fit of the number of genes involved in each process against the accuracy of each process in ROC area. The coefficient of determination (*r* ^2^) was calculated from the linear fit to be 0.39 (*p* = 0.0007). This indicates bias towards GLMs predicting for functions that have a higher number of genes involved in the function. As seen from the results in Table 1, pathways that contained fewer genes in general indicated no correlation in branch length between the genes. We would hence expect better results in some of these pathways as some of these functions become more thoroughly annotated.

For our most significantly correlated process-function of “translation” and “structural constituent of ribosome”, tests were expanded to further investigate the correlation. The size of the null distribution was increased to 10000 replicates. It is noted here that even when the replicates was increased, the *p*-value remained at 0.0, indicating that there is <0.0001 chance that the correlation seen was obtained at random. Therefore we have strong evidence to reject the null hypothesis that the correlation in gene tree phylogeny between genes labeled with GO terms “translation” and “structural constituent of ribosome” was due to random effects.

**Figure 2:**
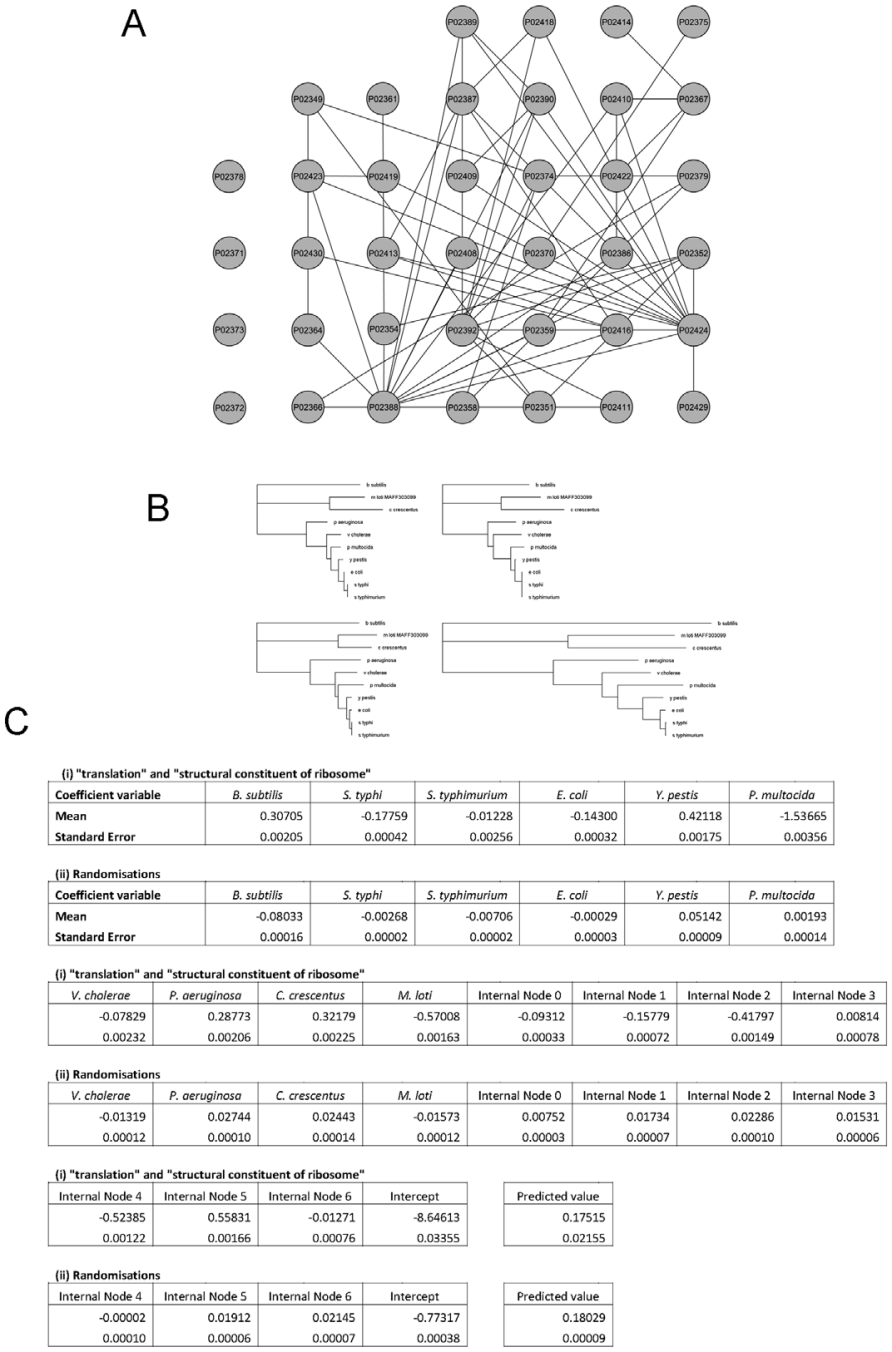
A detailed analysis of the proteins in our dataset annotated as being involved in GO process “translation” and GO function “structural constituent of ribosome”. (a) The pathway interaction network of these proteins, as shown in Cytoscape [64]. Proteins P02378, P02371, P02373 and P02372 (in the first column) contain no known physical interactions to any other proteins in our list. (b) Example gene trees of proteins from our dataset. From top left to bottom right, the trees are from gene P02386, P02410 (a protein known to physically interact with P02386), P02351 (a protein that does not interact with either of the previous genes but contributes to the pathway) and the consensus of all gene trees in our dataset not labeled with these two GO terms. (c) The models built by the GLMs for (i) the proteins labeled with the two GO terms and (ii) for the 10000 randomizations of the null distribution. The end predicted value is obtained by adding the products of each coefficient and its corresponding predictor value, and the intercept value.

We computed physical comparisons of the characteristics of proteins involved in this process, relative to other genes and processes. We tested whether the correlation in phylogeny occurs only amongst physically interacting genes, or whether correlation extends to non-interacting genes of related function. To test this, the most significantly correlated process-function pair of “translation” and “structural constituent of ribosome” was again used. The known interactions between the genes involved in this biological function were obtained from the Database of Interacting Proteins [54]. Figure 2a shows the interaction network of the proteins in our dataset labeled with these particular GO terms. Although a large number of interactions within this pathway occur between the genes, not all the genes contain an interaction with another. In fact some of the proteins contain few interactions to any of the other proteins. Yet, the correlation in gene tree branch length amongst the proteins shown here was clearly shown in the results of the leave-one-out test. Hence, it can be seen that the correlation in phylogeny between genes is not purely limited to physically interacting genes, but the correlations also exist between functionally related genes operating within the same pathway.

Figure 2b shows an example of gene trees from proteins within this pathway. From the example it can be seen that there are similarities in branch lengths between proteins functioning within the same pathway, which is not limited to only proteins that directly interact. These similarities also show distinction from other proteins, as is seen by the dissimilarities of the gene trees to the consensus tree of proteins not involved in the pathway.

Figure 2c shows the coefficients of the GLMs from modeling the correlation from these proteins. As a comparison, the average values of each coefficient from the 10000 randomizations generated is shown. It should be noted that the coefficients here model the variation in *log_e_* transformed branch lengths; therefore a large proportion of the predictor values will be negative, as branch lengths are generally small. From Figure 2c, we see that the coefficients from the actual process-function term itself differ greatly from that of the randomizations. This indicates that there is a distinction in the branch lengths of proteins in this pathway. As the intercept value and end predicted value differ between the two models, comparisons cannot be made.

Previous studies have found that for phylogenetic profiling [55] the number and choice of species affects how informative the profiles are [56], [57]. As the underlying concept of our analysis is similar to phylogenetic profiling, this may cause a bias in our results. To test whether the high correlations seen here are biased by species choice, we repeated the leave-one-out analysis. Each time it was repeated, we simulated a single taxa removal by excluding and combining columns corresponding to the branches. With the removal of taxa, the ROC area that was produced by the GLMs of “translation” and “structural constituent of ribosome” did not alter greatly from our original result. From the 10 individual species removals, the ROC areas ranged from 0.89 to 0.94, with a mean of 0.91. Therefore, the significant correlation is unlikely an effect of bias due to choice of species used in our analysis.

## Discussion

We have shown here that there are correlations between a protein's function and its gene tree branch lengths. This correlation in phylogeny is most likely attributable to the coevolution of genes that have functionally related gene products. Previous studies of inference from coevolution have focused primarily on the relationship between genes that have physically interacting gene products. We show that correlation in branch lengths extends to genes that are involved in the same functional pathway.

Hakes et al. [33] proposed the hypothesis of common selection pressures occurring on these genes to account for correlated evolutionary rates in functionally-related genes. We may also imagine that the correlations can be caused by the “contagious” propagation of mutations across the genes in the biological pathways responsible for the function. Specifically, mutations in one gene in a pathway may lead to direct compensatory mutations in a set of related genes which in turn can cause compensatory changes in other related genes, causing a cascade of mutations throughout the pathway. Alternatively, it may be that a change in the selective environment leads to changes in the selective pressure to maintain the structures of proteins involved in a given function, so that changes in substitution rates (and branch-lengths) are observed along different lineages.

In our study, we found that the correlation in branch length was particularly high in proteins involved in translation and ribosomal activities. This was most significant in proteins labeled with GO terms “translation” and “structural constituent of ribosome”. We found that the overall tree lengths of these proteins are shorter than that of other proteins (average of 2.96 in ribosomal genes and 6.30 in others). This indicates that there is an overall effect across species of purifying selection acting towards the genes coding for these proteins. This is in agreement with literature stating that strong selective pressure occurs across ribosomal and translational genes [58], [59]. An explanation for the purifying selection across these genes is that functions such as translation are crucial for an organism's basic function and therefore any changes to the protein sequence may cause disruption towards this essential pathway. It can also be seen that the degree to which purifying selection occurs differs across each species lineage. This is indicated by the coefficient values shown in Figure 2c, as each coefficient varies a different amount to what is expected on average.

In our analysis, uncovering correlation is limited to identifying genes that experience similar selective regimes. The assumption is that genes with functionally related proteins would undergo similar rates of evolution; yet it is possible for functionally unrelated genes to have undergone rate similarities. A subset of this effect is when trying to find correlations amongst genes from a common biological function where the genes are evolving neutrally or near neutrally. Gene trees of any other neutrally evolving genes will have similarities in branch length to gene trees of this function. This can confuse general classification and correlation methods into believing that these genes should be grouped within this function. This is noted as a limitation to our method but it will also confound any method that is based on identifying equivalent lineage-specific rates of evolution.

Despite the high significance seen in the correlation of some of the functions, a majority of the functions performed only marginally better than random. This suggests that the correlation in branch lengths is weak amongst genes annotated as being involved in those processes. The low correlation may be explained by a range of factors. Firstly, such biases in different processes are possibly due to issues within our dataset. A low number of genes involved in a function to train the model can lead to biased models. As mentioned previously, it is commonly known in statistics that a reasonable number of each case type relative to number of features is required to train accurate models [48]. The test in correlation showed that there was a significant correlation between the number of positive test cases in the processes and the ROC area. It is likely that this effect caused some bias in our study where functions with a larger number of genes involved are favored.

In addition, errors in the prediction can be caused by incorrect and incomplete functional annotations. Gene annotations in databases are often incomplete and contain errors. In particular for some biological processes such as gene translation, the specific functions of each gene involved in these processes are better known. Relevant processes will therefore have more complete and less erroneous annotations.

A second factor contributing to the discrepancy in correlation is natural variation in amount of selection pressure and gene constraint. Observed coevolution is an effect of similar selection pressures acting on functionally related proteins [33]. Where the selection pressures are weak, lesser correlations in substitution rates are expected. In cases where the compensatory mutations are crucial towards the coevolution, weaker structural constraints between genes with interacting products will result in less coevolution. Often mutations in amino-acid sequence cause no or small changes to the outcome of protein structure [60], [61]. While some protein interactions necessarily require coevolution, others are known to naturally have structural flexibility and can allow for changes in interaction partners without having to make changes to itself [60], [62]. Less constraint between genes would mean that correlated mutations are often inessential thereby resulting in less similarity in substitution rates. This effect is more likely in genes where the sequence of the binding surfaces is proportionally short. In these cases mutations may not have great structural modifications to the gene and compensatory mutations may not occur. As a result similarities in branches will be less evident. In addition to this, it is possible for functionally related genes to not share common patterns of evolution. As shown by Rausher et al. [40] and Lu et al. [41], genes that produce functionally related proteins can undergo different degrees of selection, as a result of relaxed constraint on some of the genes. It is possible that in our dataset certain genes have become relaxed in one or more species. As a result, there can be a lack of correlation between such genes and other genes in the pathway it is involved in.

Another explanation for weaker correlation between genes is the definitions of function provided by GO terms. GO provides a set of text vocabularies used to categorize sequences by the general attributes of their biological function. These vocabularies cannot distinguish between different pathway organizations within the function. Hence, it is often the case that functionally unrelated genes may be annotated similarly in GO.

In addition, GO terms provide no indication towards the specificity of each term. Some function terms are very specific (for example, “protein secretion by the type II secretion system”, “small GTPase mediated signal transduction”) whilst others are very general (for example, “metabolic process”, “cell cycle”).

As part of the study, we applied the same tests to the OrthoMam dataset (version 4.0) [63]. After filtering we obtained a substantial dataset containing 730 genes that were orthologous among 24 mammalian species. Results of this analysis showed no significant correlation between any genes involved in a particular function and the gene tree branch lengths for the genes. Though the data itself is abundant, the terms that were common among the genes were uninformative. For example the most abundant processes-function pairs were: “regulation of transcription, DNA-dependent” with “DNA binding”, “signal transduction” with “protein binding”, and “regulation of transcription, DNA-dependent” with “transcription factor activity”. These terms contain limited information on the underlying pathways themselves. It is likely that not all the genes function within the same pathway. The lack of correlation may also potentially be attributable to the distance between species (the overall tree length of this dataset was roughly 7.5 times shorter than that of the bacterial dataset) and the positive test case to negative test case ratio (the most abundant process-function pair only contained 5.9% of the 730 genes), which is known to cause under-fitting in model fitting.

Our study also suggests that when estimating divergence times, care should be taken because gene tree branch lengths may be biased by the function of the gene. Correlated changes in genes are more prominent in genes with gene products of related function; these will affect rate estimation if these genes are treated as multiple “independent” loci. An implication of our finding towards estimating species divergence times in comparative biology is that it is erroneous to estimate species distances using a small number of functionally-related genes. Though these effects have been to some degree recognized, they are often not considered when carrying out comparative analysis between species. A suggestion from our results is that estimating species distances should be performed using multiple loci from genes of a wide range of functions. Our findings support the suggestion made by Thorne and Kishino [15] of taking into account the correlation of genes when using multiple loci. Thorne and Kishino suggested that when estimating distances using concatenation of genes, to add parameters, models and priors which consider the correlation of substitution rates amongst the genes. Our result provides support to the use of Thorne and Kishino's techniques and as a result raises questions towards the common assumption of independence in substitution rate of gene trees.

## Supporting Information

Figure S1 Histogram of the gene tree branch lengths on the *P. multocida* branch. The length of branches is approximately distributed exponentially. The lengths of other branches on the tree also follow similar distributions.
(0.08 MB TIF)
