# Supplementary figures and images for: Covariation of Branch Lengths in Phylogenies of Functionally Related Genes
Source: PLoS One. 2009 Dec 29;4(12):e8487. doi: 10.1371/journal.pone.0008487 (PMC2793527; doi:10.1371/journal.pone.0008487)

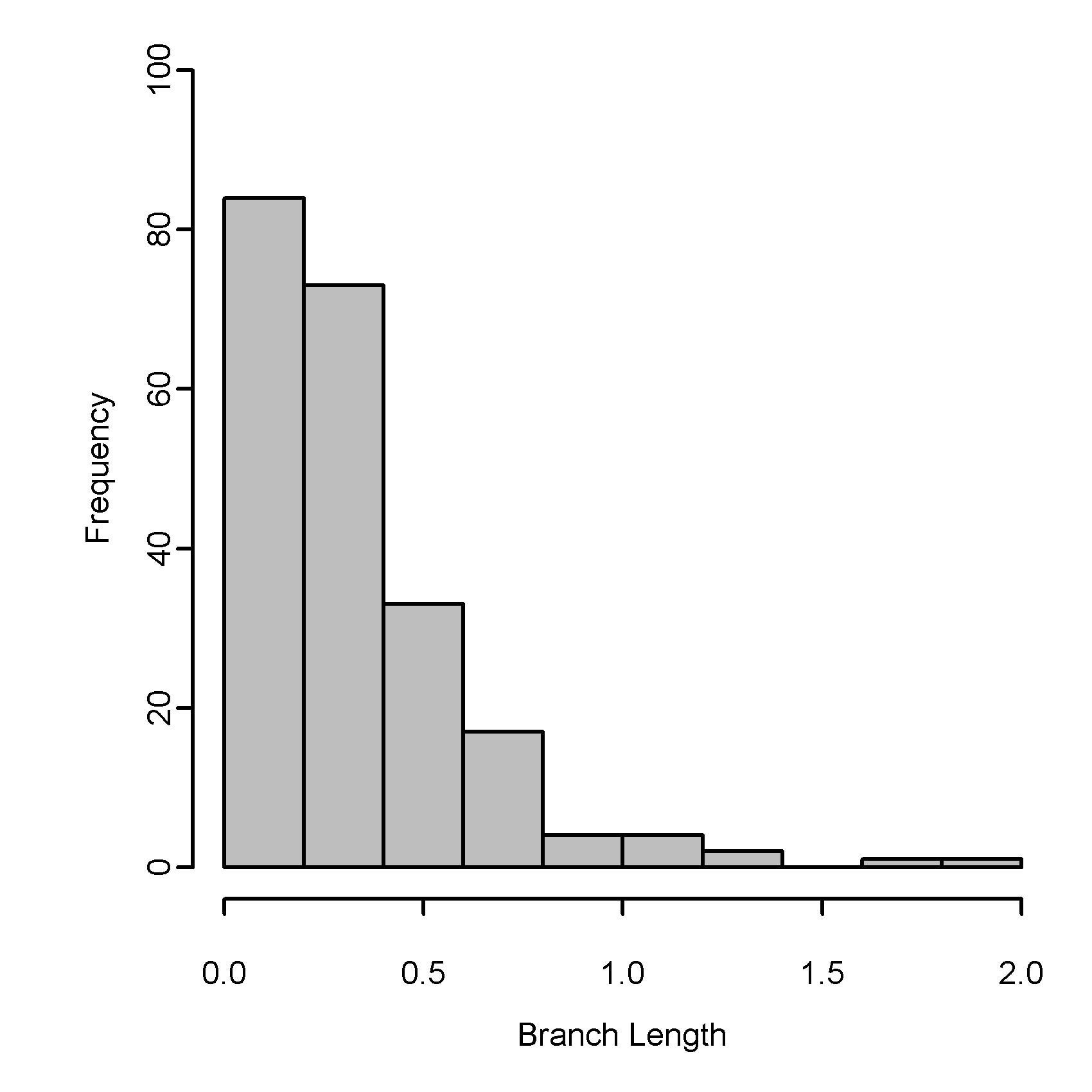

Supplement: Figure S1 — Histogram of the gene tree branch lengths on the P. multocida branch. The length of branches is approximately distributed exponentially. The lengths of other branches on the tree also follow similar distributions. (0.08 MB TIF) [file pone.0008487.s001.tif]
